# Multi-Omics Frontiers in Plant Adaptation and Growth: From Molecular Networks to Precision Breeding

**DOI:** 10.3390/plants15162519

**Published:** 2026-08-20

**Authors:** Ying Li, Zhimin Gao

**Affiliations:** National State Forestry and Grassland Administration Key Open Laboratory on the Science and Technology of Bamboo and Rattan, Institute of Gene Science and Industrialization for Bamboo and Rattan Resources, International Centre for Bamboo and Rattan, Beijing 100102, China; liying@icbr.ac.cn

## 1. Introduction

Plant growth and development unfold as a dynamic continuum, giving rise to adaptive phenotypes through the intricate interplay of genetic programs and environmental inputs. With climate change expanding and accelerating the strain on global resource security, spanning both food and timber systems, the need to decipher the molecular- and systems-level mechanisms underpinning plant adaptation has never been more pressing. Over the past decade, plant biology has undergone a transformative shift, propelled by the rapid advancement of high-throughput omics technologies. Genomics, transcriptomics, proteomics, metabolomics, and phenomics now afford unprecedented resolution for probing the systemic properties of plant tissues, pinpointing causal genes and metabolites, and reconstructing the regulatory networks that orchestrate trait emergence.

This Special Issue, entitled “Genetic and Omics Insights into Plant Adaptation and Growth,” was conceived to capture the forefront of this evolving field. The eight contributions assembled here—five original research articles and three comprehensive reviews—span a remarkably diverse array of topics, including fruit ripening, cytoplasmic male sterility, grain weight determination, transposable element dynamics, and the regulatory functions of key transcription factors and metabolic enzymes. Collectively, these studies underscore the power of multi-omics integration in elucidating complex biological processes and highlight actionable targets for molecular breeding in agriculturally and economically important species.

## 2. The Multi-Omics Landscape: Current Advances and Persistent Challenges

The integration of multiple omics layers has become a defining paradigm in plant systems biology. Although single-omics approaches have provided valuable insights, they often capture only fragments of the regulatory networks that underpin complex traits. The contributions to this Special Issue illustrate how combining transcriptomic, proteomic, metabolomic, and genomic data can yield a more holistic view of the molecular dynamics governing plant development and environmental adaptation.

A recurring theme across both the original research and review articles in this collection is the critical role of temporal resolution in developmental studies. Chen et al. (2026) demonstrated that knockout of the cell wall invertase inhibitor *SlINVINH1* accelerates tomato fruit ripening by 2–3 days, with transcriptomic profiling revealing enrichment of differentially expressed genes in pathways associated with ripening and sugar metabolism [1]. Beyond identifying a novel negative regulator of fruit ripening, this work offers a genetic strategy for concurrently enhancing fruit sweetness and accelerating maturation—a dual improvement of considerable practical value.

In a complementary study, Xuan et al. (2025) performed integrated transcriptomic and proteomic analyses of wheat grain development and pinpointed 7 days post-anthesis as a critical developmental window during which differential regulation of carbohydrate biosynthesis determines final grain weight [2]. Through weighted gene co-expression network analysis (WGCNA) and K-means clustering, the authors identified *TaYAK1-2D* as a positive regulator of grain weight, a finding further supported by mutational validation [2]. This study exemplifies how multi-omics integration can delineate temporally sensitive developmental phases and prioritize high-confidence targets for breeding programs.

The two companion studies on cytoplasmic male sterility (CMS) in tobacco further underscore the power of integrated approaches [3,4]. By combining transcriptomic, metabolomic, and histological analyses, these investigations revealed that stamen identity defects in CMS K326 are associated with concurrent disruptions in energy metabolism and auxin homeostasis [3,4]. Specifically, metabolomic profiling uncovered reduced levels of glycolytic intermediates and auxin deficiency, while transcriptomic analysis detected aberrant expression of floral organ identity genes, including *WUSCHEL*, *GLOBOSA*, and *SUPERMAN* [4]. Collectively, these findings provide multi-omics-level evidence that disrupted nuclear–cytoplasmic interactions precipitate metabolic and hormonal imbalances that jointly impair stamen development.

Despite these advances, several challenges remain. As emphasized in the review on bamboo shoot bud development, traditional approaches using whole shoot buds or mixed tissues obscure cellular and tissue heterogeneity [5]. The authors identify four key bottlenecks: tissue homogenization masking cellular heterogeneity, loss of spatial positional information, technical difficulties in applying single-cell technologies due to sample preparation constraints, and the unresolved coupling between chromatin accessibility and transcriptional regulation [5]. Importantly, these limitations are not confined to bamboo but reflect broader obstacles facing plant developmental biology as a whole.

## 3. Emerging Frontiers: Single-Cell Resolution, Spatial Context, and Epigenetic Regulation

Recent breakthroughs in single-cell and spatial transcriptomics are set to transform plant developmental biology [6,7]. These technologies resolve cellular heterogeneity while preserving spatial context [8]. For example, a recent *Arabidopsis* atlas—integrating single-nucleus and spatial transcriptomic data across ten developmental stages—has demonstrated this potential [9]. It revealed remarkable molecular diversity across cell types and states. It also uncovered conserved transcriptional signatures among recurrent cell types, alongside organ-specific heterogeneity [9]. Single-cell transcriptomic studies in *Arabidopsis* have laid the foundation for plant single-cell genomics [6,7,10], and subsequent studies have generated single-cell atlases for rice roots [11,12], leaves, florets and inflorescence meristems [13], wheat spikes [14], maize shoot apex [15], and cotton fibers [16]. For perennial woody species, single-cell transcriptomic maps have also been established for poplar stems [17].

The bamboo shoot bud review presents a compelling vision for applying these technologies to non-model species [5]. The authors propose a core strategy. This strategy centers on constructing a single-cell resolution, spatially resolved, multi-omics integrated framework. Such an approach would address a fundamental question. How do spatially distinct meristematic populations coordinate differentiation and maturation? These include vertically arranged procambium in the culm wall and horizontally arranged procambium at nodal diaphragms. Applying spatial transcriptomics to bamboo shoot buds remains technically challenging. Tissue complexity and the need for optimized permeabilization protocols are major hurdles. Nevertheless, this approach promises to reveal the molecular basis of bamboo’s unique developmental patterns [5]. It may also provide key targets for genetic improvement of shoot quality and mechanical properties.

Recent progress in bamboo single-cell research has been achieved, with single-cell transcriptomic atlases generated for bamboo roots [18], single-internode stems, intercalary meristems [19], and shoot buds. However, owing to technical limitations and the lack of mature genetic transformation systems for bamboo, current studies remain largely confined to cell-type classification and transcriptional characterization [5]. Spatial omics technology, which captures cellular spatial information while preserving positional context [20,21], has been applied to a limited number of plants, including wheat [14], poplar [17], and bamboo [19], but widespread adoption has not yet been achieved due to challenges such as tissue complexity and the need for optimized permeabilization protocols [5].

Epigenetic regulation represents another frontier being increasingly integrated into multi-omics frameworks. The review on phenylalanine ammonia-lyase (PAL) covers this comprehensively [22]. It describes a multi-layered regulatory network governing this core metabolic enzyme. This network includes transcriptional control, post-translational modifications, epigenetic regulation, and functional crosstalk with phytohormones. PAL acts as a “metabolic valve.” It connects primary aromatic amino acid metabolism to the phenylpropanoid pathway. As such, it is a central determinant of carbon flux redistribution between growth and defense. Understanding how epigenetic modifications—especially DNA methylation and histone modifications—influence PAL expression and activity is critical. This is important across developmental stages and in response to environmental stress. Such knowledge will help optimize carbon allocation in crops [22].

Chromatin accessibility—reflecting the extent to which *cis*-regulatory elements such as promoters, enhancers, and silencers are physically accessible—arises from the combined effects of nucleosome occupancy, topological configuration, transcription factor binding, and chromatin remodeling complexes [23]. In transcriptionally active regions, nucleosome arrays are relatively loosely arranged, and intervening DNA sequences frequently interact with regulatory proteins. Dynamic changes in chromatin accessibility landscapes broadly reflect their regulatory capacity and serve as key determinants of chromatin organization and function [23,24]. Recent advances in plant single-nucleus isolation techniques have revealed extensive single-cell accessibility diversity across various plant tissue types. Studies on rice radicle [11] and wheat embryo [25] development have integrated single-cell RNA sequencing, chromatin accessibility assays, and histone modification profiling to dissect cell type specification processes and elucidate the regulatory networks underlying cell fate determination.

The cotton transposon dynamics study offers a striking example [26]. It shows how genomic structural variation shapes phenotypic diversity. The authors analyzed 28 cotton genomes. They constructed a pan-TE-related structural variation map. Their findings demonstrate that the Tekay subclass of the *Gypsy* superfamily is a major driver of *Gossypium* genome evolution [26]. Notably, two burst events occurred in the At-subgenome. Critically, 334 transposon-related variations showed statistically significant associations with 10 key phenotypic traits. Among these, 164 affected yield components and 170 determined fiber quality [26]. This work illuminates the evolutionary significance of transposable element dynamics. It also provides novel functional markers and potential editing targets for cotton molecular breeding. Recent advances in transposon annotation and pangenome analysis have further revealed that transposons are major targets of epigenetic silencing [27,28]. Their distributions are highly structured along plant chromosomes [27]. Additionally, the capture and repurposing of host genes by transposable elements represents another mechanism. This mechanism drives genome plasticity and phenotypic innovation [28].

The bamboo shoot bud review also emphasizes the importance of understanding bamboo’s unique developmental biology [5]. China has rich bamboo resources, with Moso bamboo (*Phyllostachys edulis*) being the most economically important species [29]. Bamboo shoot bud development directly determines the eating quality of the shoots and the properties of bamboo materials [5,9,29,30]. Traditional methods using whole shoot buds or mixed tissues obscure cellular and tissue heterogeneity, limiting our mechanistic understanding of this process [5]. Cytological studies have revealed that shoot bud development is characterized by diverse meristem types, strict spatiotemporal regulation, pronounced cellular heterogeneity, and a highly dynamic chromatin structure [31,32]. Transcriptome and miRNAome analyses have identified the miRNA-mRNA modules involved in biological pathways, including plant hormone signal transduction, carbohydrate metabolism, and microtubule movement, which regulate key processes such as cell wall synthesis and lateral root growth in bamboo shoot buds [33].

## 4. Translational Opportunities and the Integration of Systems Biology

Translating omics-derived insights into breeding applications remains a central challenge. The KNOX1 transcription factor review critically examines this bottleneck [34]. It identifies several key constraints. These include fragmented regulatory networks, recalcitrant transformation systems in woody perennials, and a near-complete absence of multi-environment field validation. The authors propose four strategic priorities [34]: first, construct spatiotemporal expression atlases using single-cell and spatial transcriptomics; second, employ tissue-specific and promoter-engineered CRISPR/Cas9 gene editing to circumvent pleiotropic penalties; third, conduct cross-species comparative evo-devo analysis of lineage-specific innovations; fourth, implement integrated field trials that assess genotype-by-environment interactions and trait stability.

The concept of “closing the loop” between multi-omics discovery and field validation is gaining traction. This idea is increasingly being embraced by the broader plant science community. Recent reviews have articulated a vision for the AI-driven integration of multi-omics and phenomics. This approach could transform crop improvement into a predictive engineering discipline [35]. The framework involves deploying digital twins for in silico stress simulation. It also uses generative AI for de novo molecular design. Experimental outcomes iteratively refine computational models [35]. These approaches are still nascent. Nevertheless, they hold promise for accelerating the design–build–test–learn cycle. They also help bridge the gap between laboratory discovery and field deployment.

The systems biology perspective emphasizes that plant physiology emerges from dynamic interactions. These interactions occur among genes, proteins, metabolites, and environmental cues. They form multilayered systems. Such systems cannot be fully understood through reductionist approaches alone [36,37]. Representative case studies illustrate how multi-omics integration can bridge scales. Examples include single-cell transcriptomic atlases in *Arabidopsis* [6,7,10,11], nitrogen-use-efficiency modeling, and omics-guided genome editing in cereals. These studies connect laboratory-scale discovery with field-level validation. The wheat grain weight study in this Special Issue exemplifies this translational pathway [2]. The authors identified a favorable haplotype (*TaYAK1-2D-Hap2*). They also developed a practical InDel marker for molecular breeding [2].

## 5. Future Directions: Towards Predictive and Integrative Plant Biology

Looking ahead, several directions warrant particular attention.

First, applying single-cell and spatial omics to non-model and horticultural species will be essential. This will help translate mechanistic insights from model systems to agriculturally relevant contexts. The bamboo shoot bud review provides a roadmap for this transition [5]. It emphasizes the need for species-specific protocol optimization. It also calls for standardized nomenclature and functional validation platforms. As highlighted in the review, the application of single-cell technology to bamboo shoot buds currently faces challenges such as a weak foundational omics reference base, a lack of spatial information, difficulties in cell dissociation, insufficient resolution of developmental dynamics, inconsistent nomenclature systems, and a lack of functional validation platforms [5]. This will require bamboo-specific cell dissociation and nuclei isolation protocols, a single-cell reference atlas, and the integration of spatial transcriptomics with in situ validation.

Second, integrating multi-omics data with environmental variables is a key priority. Systems biology approaches will enable predictive modeling of genotype-by-environment interactions. The PAL review highlights the complexity of combined stresses [22]. For instance, drought plus heat, or drought plus pathogen attack, induce unique sets of stress-responsive genes. These genes are not activated under individual stress conditions. Understanding how plants integrate multiple stress signals is critical, as is understanding how they prioritize carbon allocation among competing demands. This knowledge is essential for developing climate-resilient crops.

Third, the convergence of AI with multi-omics and phenomics is reshaping plant science. This represents a fundamental restructuring of the field. It moves beyond correlation-based analysis and toward causal, generative engineering frameworks [35]. Deep representation learning offers powerful tools. These include transformer architectures and graph neural networks. They can decode the non-linear dynamics of genotype–environment–management interactions. Such interactions elude linear statistical models. However, the black-box nature of deep learning models presents interpretability challenges. These must be addressed through explainable AI and causal inference approaches.

Fourth, the role of transposable elements is increasingly recognized. They drive genome evolution and phenotypic variation. Yet their functional integration into regulatory networks remains poorly understood. Recent advances in long-read sequencing have provided new insights. Transposons are major targets of epigenetic silencing [27]. Their distributions are structured along plant chromosomes [27]. The cotton study in this Special Issue demonstrates that transposon-related structural variations can serve as valuable markers for agronomic traits [26]. This suggests that pan-TE maps could become standard tools in crop genomics.

Finally, translating omics-derived candidate genes and regulatory variants into breeding applications remains a central challenge. This includes marker-assisted selection, genomic selection, and genome editing. Success requires close collaboration between basic researchers, breeders, and agricultural stakeholders. The KNOX1 review emphasizes that constitutive overexpression often triggers deleterious pleiotropic effects. This necessitates tissue-specific and promoter-engineered approaches [34]. Developing efficient functional validation platforms will be essential. These include transient transformation, virus-induced gene silencing, and CRISPR-based gene editing. Such tools will accelerate the translation of fundamental discoveries into improved crop varieties.

## 6. Conclusions

The contributions to this Special Issue showcase remarkable progress in applying omics technologies to plant biology. These studies address growth, development, and adaptation. They demonstrate the power of integrating multiple molecular layers. This integration generates a more complete picture of plant biology. Examples range from dissecting fruit ripening regulatory networks [1] to characterizing transposable element dynamics in genome evolution [26].

At the same time, these studies highlight persistent challenges. Several key issues remain. These include the need for single-cell and spatial resolution, alongside the importance of epigenetic regulation. Furthermore, the complexity of genotype-by-environment interactions requires attention. Finally, the gap between laboratory discovery and field application must be bridged.

Looking ahead, exciting opportunities are emerging. The convergence of multi-omics technologies, systems biology approaches, and artificial intelligence is transformative [35]. These tools offer unprecedented potential. They not only deepen our understanding of plant adaptation but also help develop crops that thrive in a changing climate.

Embracing these integrative frameworks is essential, fostering collaboration across disciplines. In this way, the plant science community can accelerate the critical transition from descriptive analysis toward predictive, design-driven biology.

The research presented in this Special Issue marks an important step in bridging laboratory discovery and breeding applications. It provides substantive findings, as well as a number of conceptual frameworks. These will inspire future investigations at the intersection of genetics, omics, and plant adaptation.

## Data Availability

No new data were created or analyzed in this study. Data sharing is not applicable to this article.

## Abbreviations

The following abbreviations are used in this manuscript:

WGCNA	weighted gene co-expression network analysis
CMS	cytoplasmic male sterility
PAL	phenylalanine ammonia-lyase
